# Measurement of Peripheral Nerve Magnetostimulation Thresholds of a Head Solenoid Coil Between 200 Hz and 88.1 kHz

**DOI:** 10.1109/JTEHM.2025.3570611

**Published:** 2025-05-15

**Authors:** Alex C. Barksdale, Natalie G. Ferris, Eli Mattingly, Monika Śliwiak, Bastien Guerin, Lawrence L. Wald, Mathias Davids, Valerie Klein

**Affiliations:** MITDepartment of Electrical Engineering and Computer Science Cambridge MA 02139 USA; Martinos Center for Biomedical Imaging Charlestown MA 02129 USA; Harvard Graduate Program in BiophysicsHarvard University 1812 Cambridge MA 02138 USA; Harvard-MIT Division of Health Sciences and Technology Boston MA 02139 USA; Harvard Medical School Boston MA 02115 USA

**Keywords:** High frequency magnetostimulation, magnetic particle imaging (MPI), magnetic resonance imaging (MRI), MRI gradient safety, peripheral nerve stimulation

## Abstract

Magnetic fields switching at kilohertz frequencies induce electric fields in the body, which can cause peripheral nerve stimulation (PNS). Although magnetostimulation has been extensively studied below 10 kHz, the behavior of PNS at higher frequencies remains poorly understood. This study aims to investigate PNS thresholds at frequencies up to 88.1 kHz and to explore deviations from the widely accepted hyperbolic strength-duration curve (SDC).PNS thresholds were measured in the head of 8 human volunteers using a solenoidal coil at 16 distinct frequencies, ranging from 200 Hz to 88.1 kHz. A hyperbolic SDC was used as a reference to compare the frequency-dependent behavior of PNS thresholds.Contrary to the predictions of the hyperbolic SDC, PNS thresholds did not decrease monotonically with frequency. Instead, thresholds reached a minimum near 25 kHz, after which they increased by an average of 39% from 25 kHz to 88.1 kHz across subjects. This pattern indicates a significant deviation from previously observed behavior at lower frequencies.Our results suggest that PNS thresholds exhibit a non-monotonic frequency dependence at higher frequencies, diverging from the traditional hyperbolic SDC. These findings offer critical data for refining neurodynamic models and provide insights for setting PNS safety limits in applications like MRI gradient coils and magnetic particle imaging (MPI). Further investigation is needed to understand the biological mechanisms driving these deviations beyond 25 kHz.***Clinical impact***—These findings call for further basic research into biological mechanisms underlying high frequency PNS threshold trends, and supports refinement of safety guidelines for MRI and MPI systems for clinical implementation.

## I. Introduction

Magnetic resonance imaging (MRI) gradient coils and magnetic particle imaging (MPI) drive coils generate time-varying magnetic fields (B-fields). These time-varying B-fields induce electric fields (E-fields) in patients that can cause peripheral nerve stimulation (PNS) [2]. PNS is typically perceived as mild muscle twitches or tingling, but higher stimulus amplitudes can cause intolerable or painful sensations [3], [4]. The International Electrotechnical Commission (IEC) 60601-2-33 standard places limits on the maximum switching speed of MRI gradient fields to minimize PNS and ensure subject comfort and safety [5]. PNS limits for a given MRI gradient coil design are typically determined from PNS measurements in a small cohort of healthy subjects. Magnetostimulation is well characterized experimentally for frequencies below 10 kHz [4], [6], [7], [8], [9], [10], [11], [12], [13], but the data is sparse at higher frequencies. The relationship between PNS B-field threshold amplitude and frequency can be described by the hyperbolic strength-duration curve (SDC), which predicts a monotonically decreasing PNS threshold with increasing frequency [14]. Some recently developed MRI gradient systems are operating at frequencies >10 kHz for increased imaging speed, or reduced acoustic noise [8], [9], [15]. MPI drive fields for excitation typically operate at 25 kHz, and lately there have been efforts to develop instrumentation [16] to enhance sensitivity for MPI at even higher frequencies. It is therefore necessary to characterize PNS limits at frequencies above 10 kHz to ensure safe operation of MRI and MPI systems in human subjects.

Three studies have reported deviations between measured PNS thresholds and the hyperbolic SDC at frequencies above 25 kHz [7], [17], [18]. Schmale et al. measured PNS thresholds at four frequencies between 24 and 162 kHz produced by a solenoid and a saddle coil in the abdomen of five healthy male volunteers [17]. For both coils, they observed that the average PNS threshold across subjects increased with frequency, directly contradicting the hyperbolic SDC. Weinberg and Stepanov et al. measured PNS thresholds in the forearm of 26 healthy volunteers at five frequencies between 2 kHz and 183 kHz [18]. They also observed increasing PNS thresholds for frequencies above 25 kHz, again contradicting the hyperbolic SDC. However, both studies only measured few frequencies, and the data displayed high variability (up to 2X) across subjects, making it difficult to draw definitive conclusions. Saritas et al. measured PNS thresholds in the arm of 26 subjects using a solenoid coil at frequencies of 10.3, 25.5, and 50 kHz [7]. On average, they observed a 6% increase in thresholds from 25.5 kHz to 50 kHz. Some variance between subjects was noted, with one subject displaying a 10% reduction and another a 17% increase from 25.5 kHz to 50 kHz. Another study provided preliminary measurements of PNS in a head MPI solenoid at 24 kHz in three subjects [19], [20], suggesting that PNS may limit drive coil amplitude, and thus limit sensitivity in developing MPI scanners. In short, the data is very sparse, limited and highly variable and indicates that the hyperbolic SDC may not be adequate to describe PNS thresholds beyond 25 kHz. However, existing measurements do not provide definitive evidence thus calling for additional measurements to be made.

In this work, we perform PNS magnetostimulation experiments in the heads of 8 healthy human subjects at 16 frequencies between 200 Hz to 88.1 kHz using sinusoidal stimulus waveforms. We use a human head solenoid [21] designed for our recently developed and constructed human scale fMPI scanner [22]. We compare our measurements to the hyperbolic frequency relationship predicted by the hyperbolic SDC. These measurements are helpful to set practical safety limits in human MPI systems using similar drive coil geometry [19].

## II. Methods

### A. Magnetostimulation Coil and Capacitor Bank Design

**FIGURE 1.**
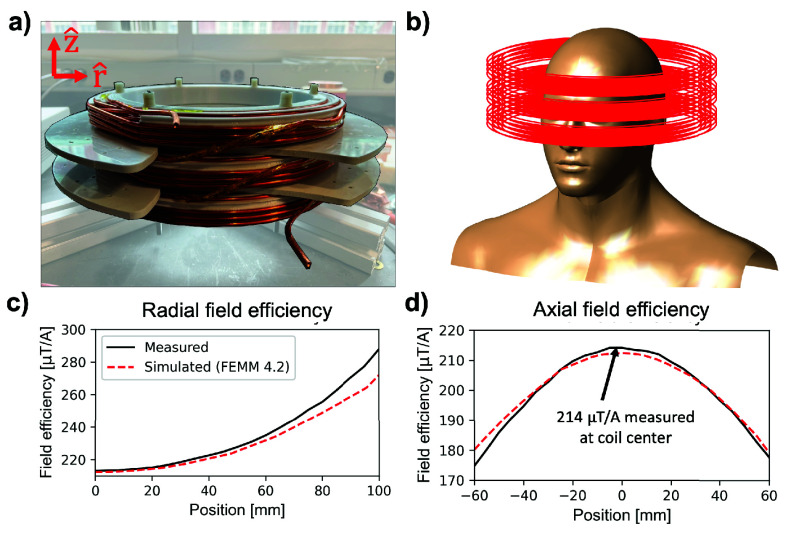
(a) Image of the solenoidal coil used in human head PNS studies. (b) Illustration of coil winding position relative to the subject’s head. (c), (d) Comparison of measured field efficiencies using a 3-axis hall magnetometer and field mapping robot and simulated field efficiencies using FEMM 4.2. Field efficiencies were evaluated radially (at z=0) in (c) and axially (at r=0) in (d).

**FIGURE 2.**
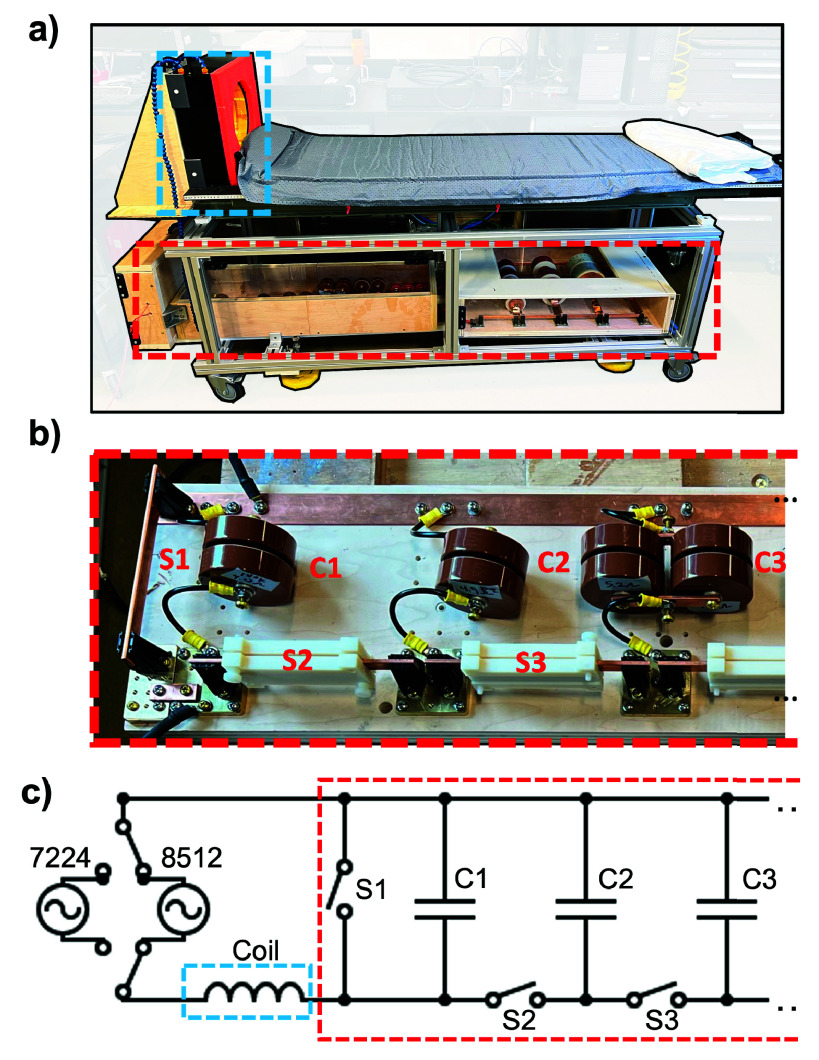
(a) Image of patient bed used for human head PNS studies, with coil mounted (blue dashed box) and reconfigurable capacitor bank (red dashed box). (b) Image of constructed capacitor bank. (c) Circuit schematic detailing amplifier connection and switchable capacitor bank. Switch S1 allows for bypassing the capacitor bank to operate in untuned configuration. Switches S2, S3...connect additional capacitors in parallel, enabling rapid changing of LC tuning for human studies.

We constructed a solenoidal head coil based on our previously described human MPI scanner drive coil design [21]. An image of the coil windings is shown in Fig. 1a. The coil has three layers wound with hollow Cu wire (4 mm outer diameter, 2 mm inner diameter), with a total of 54 turns. The coil has a length of 11 cm, an inner winding diameter of 27 cm, and is wound on a plastic coil former with 24 cm inner diameter. We employed a field mapping robot and a Metrolab THM1178 (Metrolab Technology SA, Geneva, Switzerland) three-axis field magnetometer to measure the field efficiency $\mathrm {B_{eff}}$ of the coil (B-field per Ampere of current) and compared the measurement to the efficiency computed using FEMM 4.2 [23]. We drove the coil in either an “untuned” or “tuned” configuration. The untuned configuration connected the coil inductance directly to the output of the RF power amplifier. The untuned configuration was used at 6 frequencies (200, 300, 400, 500, 600, and 700 Hz). The thresholds measured at the 10 frequencies above 1 kHz (specifically, at 1.76, 2.59, 4.04, 8.05, 16.9, 25.3, 35.4, 49.0, 66.7, and 88.1 kHz) used a capacitor in series with the coil to cancel the loads reactive impedance. This resonant drive allowed an amplifier with limited output voltage to reach sufficient field amplitudes to achieve PNS at frequencies above 1 kHz. Resonance was achieved using a configurable (rapidly switchable) capacitor bank. Fig. 2 shows a circuit schematic of the capacitor bank in series with the coil. Bus bar clips were used as manual switches that could rapidly connect and disconnect circuit components. Switch S1 in open position bypasses the capacitor bank for untuned operation. When S1 is closed, switches S2, S3, etc., can be to add capacitors in parallel to decrease the resonant frequency of the circuit. We use Celem C500T and C700T capacitors for the 1.76–8.05 kHz resonant conditions, and TDK FHV-3AN capacitors for resonant frequencies from 16.9 kHz and above. The total capacitor values for each configuration of the capacitor bank measured using an Agilent 4263B LCR meter (Agilent Technologies, Santa Clara, CA, USA) at 20 kHz were 3.73, 6.54, 12.1, 23.3, 45.6, 101, 453, 1870, 5020, and 14400 nF. The switchable capacitor bank, together with the head coil, was mounted on a patient table as shown in Fig. 2.

### B. Stimulus Waveforms

#### 1) Waveform Generation and Acquisition

We used a NIDAQ USB 6363 X-Series (National Instruments, Austin, TX, USA) to generate sinusoidal pulses with 256 cycles, and thus different frequency waveforms vary in total duration. We modulated the pulses using an exponential rampup envelope with a time constant of 25 cycles for each frequency as described below. This ensured that each sinusoidal pulse reached an asymptotic plateau amplitude (steady state) after a similar number of cycles. For frequencies below 25 kHz, we drove the coil using an AE Techron 8512 amplifier (AE Techron, Elkhart, IN, USA). For frequencies above 25 kHz, we used two high-frequency AE Techron 7224 amplifiers in a push-pull configuration (Fig. 2c). Amplifiers were configured in voltage-controlled mode. A Rogowski coil monitored the current of the Techron 7224 amplifiers, and the internal BNC current monitor was used for the Techron 8512. A 50:1 Tektronix P5200 high voltage differential probe (Tektronix, Beaverton, OR, USA) measured the voltage in the circuit. A Rohde & Schwarz RTB2004 digital oscilloscope (Rohde & Schwarz, Columbia, MD, USA) collected all measured current and voltage signal traces for subsequent processing.

#### 2) Pulse Shaping

In the tuned operating mode, the current waveform in response to a sinusoidal step voltage input at the resonant frequency is well approximated by:

\begin{equation*} i(t) = I_{0} \left ({{1 - \exp \left ({{-\frac {t}{\tau _{nat}}}}\right )}}\right ) \sin (2\pi f t + \phi ) \tag {1}\end{equation*}

The natural time constant $\mathrm {\tau _{nat} = 2L/R}$ of the rampup envelope varies across frequencies due to varying series resistances of each capacitor configuration resulting in different total circuit resistances R of each circuit. PNS is known to depend on the waveform shape of the stimulating pulse [24], [25]. We therefore modulated the input voltage waveforms to enforce similar rampup time constants for each frequency to minimize differences in waveform shape and their impact on the PNS threshold. The modulated voltage waveforms for each tuned condition were of the form (see Supplementary Information, Pulse Shaping):

\begin{equation*} v_{tuned}(t) = V_{0} \left ({{1 - \frac {\tau _{des} - \tau _{nat}}{\tau _{des}} \exp \left ({{-\frac {t}{\tau _{des}}}}\right )}}\right ) \sin (2\pi f t) \tag {2}\end{equation*}

where $\mathrm {V_{0}}$ is the steady state voltage amplitude in volts, $\mathrm {\tau _{des}}$ is the desired (enforced) time constant in seconds, and f is the frequency of the stimulus waveform in Hz. The resulting current waveforms are well approximated by:

\begin{equation*} i(t) = I_{0} \left ({{1 - \exp \left ({{-\frac {t}{\tau _{des}}}}\right )}}\right ) \sin (2\pi f t + \phi ) \tag {3}\end{equation*}

We applied the following voltage waveform to achieve similar current waveforms of the form (3) in the untuned conditions, which have no natural rampup modulation:

\begin{equation*} v_{\text {untuned}}(t) = V_{0} \left ({{1 - \exp \left ({{-\frac {t}{\tau _{des}}}}\right )}}\right ) \sin (2\pi f t) \tag {4}\end{equation*}

Note that in (3), $\mathrm {\phi \approx 0}$ for tuned conditions when operating at the resonant frequency, and $\mathrm {\phi =\tan ^{-1}(2\pi fL/R)}$ for untuned conditions.

We performed a resonance characterization before each subject session to determine $\mathrm {\tau _{nat}}$ to compute the voltage envelopes for tuned configurations. Specifically, we performed a frequency sweep of 20 points over a 400 Hz window around the expected resonance to determine the resonance frequency, $\mathrm {f_{res}}$, to within 20 Hz. The longest natural ramp-up time constant among our tuned configurations was $\mathrm {\tau _{nat}\approx 631 \mu \text {s}}$ at 35.4 kHz, corresponding to 22.3 sinusoidal cycles of this frequency. We thus chose a target time constant $\mathrm {\tau _{des} = 25/f}$ for all conditions, corresponding to 25 cycles. This ensured that $\mathrm {\tau _{des}> \tau _{nat}}$ for all tuned frequency conditions, while minimizing the voltage amplitude requirements of our amplifiers based on (2) to achieve maximum fidelity current waveform shapes.

#### 3) Waveform Analysis

We fitted the measured current waveforms using (3) to estimate the experimental current amplitude, time constant, frequency, and phase for each applied stimulus. We also fitted the steady state portion of the voltage waveforms using a simple sinusoid. The equivalent series resistance at resonance is computed as the ratio $\mathrm {R_{series}} = \mathrm {V_{0,\mathrm {fit}}} / \mathrm {I_{0,\mathrm {fit}}}$. Supplementary Table S1 lists the values for $\mathrm {f_{res}}$, $\mathrm {\tau _{fit}}$, and $\mathrm {R_{series}}$ for all experimental conditions.

### C. PNS Threshold Measurements

#### 1) Subject Training and Threshold Titrations

We measured PNS thresholds in 8 healthy adult volunteers (4 males, 4 females, average age $36~\pm ~15$ years (min: 25, max: 60 years), weight $79.4~\pm ~21.4$ kg (min: 55.8, max: 124.7 kg), height $1.73~\pm ~0.10$ m (min: 1.60, max: 1.85 m) and BMI $26.4~\pm ~6.2$ (min: 21.1, max: 40.6)) under approval of the IRB (Protocol# 2021P000349) of the Massachusetts General Hospital. Written and informed consent was obtained from all participants prior to their inclusion in the study, and all methods were carried out in compliance with relevant regulations and institutional policies. Subjects were placed on the table in supine position with their eyebrows at coil center. The subject response to each B-field pulse was recorded with a push button, indicating whether the subject felt any PNS sensation. Subjects wore hearing protection and were trained to differentiate any noise from mild coil vibrations at audible frequencies ( $\leq 8$kHz) from PNS sensations during a training session prior to the measurements. Above 8 kHz, there was no discernible coil vibration over the ranges used for PNS measurements. During this training, we played a series of pulses at 400 Hz and 16.9 kHz whose amplitude we slowly incremented until PNS was perceived by the subject. This training allowed us to obtain an initial estimate of the subject’s PNS thresholds, and helped the subject understand the sensations they may experience during PNS. During the measurements, we slowly ramped up the field amplitude in steps of ~5 mT (for frequencies >1 kHz) or in steps of ~10 mT (frequencies <1 kHz) until the subject reported a sensation (initial PNS threshold estimate). After this coarse search, we titrated the threshold with a finer sampling step size equal to 1/80th of the initial PNS threshold. The PNS threshold estimates for each frequency were obtained and continuously updated by fitting a sigmoidal function to the binary subject responses ( $\mathrm {g_{fit}(B)=1}$: subject reported PNS at the B-field amplitude B, $\mathrm {g_{fit}(B)=0}$: subject did not report any PNS):

\begin{equation*} g_{fit}(B) = \left ({{1 + \exp \left ({{-\frac {B - B_{th}}{B_{width}}}}\right )}}\right )^{-1} \tag {5}\end{equation*}

where $\mathrm {B_{th}}$ is the PNS threshold, and $\mathrm {B_{width}}$ is a width parameter related to the sharpness of the transition from non-stimulating to stimulating field amplitudes, and thus related to the uncertainty of the threshold estimate for a specific subject, frequency. Overall, each subject session lasted around 1.5 hours. We further assessed the test-retest variability of our measurements in five subjects by repeating threshold titrations at 1.76 kHz and 25.3 kHz at different points over the course of the experiment. We also recorded the stimulation site and type of sensation (e.g., tingling or pinching) reported by the subjects at each frequency.

#### 2) Average PNS Thresholds Across All Subjects

At three frequencies, some subjects either did not report any stimulation or only experienced stimulation at the amplifier’s maximum current output due. This occurred in 2 out of 8 subjects at 600 Hz, 5 out of 8 subjects at 700 Hz, and 5 out of 8 subjects at 88.1 kHz. Not accounting for this missing data can introduce bias in threshold average estimates. We corrected for this effect by assuming that threshold data for a single frequency are normally distributed among subjects, which allowed us to fit the cumulative stimulation data to the known cumulative density function for a Gaussian:

\begin{equation*} F(B) = \frac {1}{2} + \frac {1}{\sqrt {\pi }} \int _{0}^{B} \exp \left ({{-\frac {(B - \bar {B}_{th})^{2}}{2\sigma ^{2}}}}\right ) \, dB~ \tag {6}\end{equation*}

This fitting process allows us to estimate the mean and standard deviation of the data and implicitly account for missing data due to power amplifier limitations by assuming a normal distribution of thresholds across subjects.

#### 3) Hyperbolic Strength-Duration Curve

The hyperbolic SDC for sinusoidal stimuli predicts a monotonically decreasing stimulation threshold, $\mathrm {B_{th}}$, with increasing stimulus frequency, f [14]:

\begin{equation*} B_{th}(f) = B_{rheo} \left ({{1 + \frac {1}{2\tau _{chron} f}}}\right ) \tag {7}\end{equation*}

$\mathrm {B_{rheo}}$ is the B-field rheobase in Tesla (i.e., the lowest threshold asymptote reached at high frequencies), and $\mathrm {\tau _{chron}}$ is the chronaxie time constant in seconds (i.e., the time at which the threshold is twice the rheobase). We compare our PNS measurements to the hyperbolic SDC by fitting (6) to the variation of average thresholds with frequency.

#### 4) Effective Pulse Duration

In MRI, trapezoidal waveforms are commonly employed in gradient pulse sequences. The pulse duration, $\mathrm {t_{pulse}}$, of a MRI gradient pulse is defined as the time duration between the plateaus of the MRI waveform [6]. The IEC 60601-2-33 proposes effective pulse duration ( $\mathrm {t_{eff}}$) as a metric to facilitate comparisons between amplitudes of arbitrary gradient waveform shapes [5], such a comparing the sinusoidal waveforms in this study to trapezoidal waveforms commonly used in MRI. $\mathrm {t_{eff}}$ is defined as the difference between amplitudes of the first two waveform peaks, divided by the maximum derivative of the waveform over this interval. For a trapezoidal waveform, $\mathrm {t_{eff}=t_{pulse}}$. However, for a sinusoidal waveform of frequency f:

\begin{equation*} t_{eff}=\frac {2}{\pi }t_{pulse}= \frac {1}{\pi f} \tag {8}\end{equation*}

We present measured PNS thresholds versus $\mathrm {t_{eff}}$ in addition to frequency to facilitate comparison of our measurements against arbitrary waveform shapes, following the IEC recommendation.

#### 5) Normalizing Pulse Duration

Saritas et al. investigated the relation between the PNS threshold and total duration of a sinusoidal pulse [26]. They found that this relationship can be described by the following function:

\begin{equation*} B_{norm}(T_{pulse}) = 1 + \alpha \exp \left ({{-\left ({{\frac {T_{pulse}}{\beta }}}\right )^{\gamma }}}\right ) \tag {9}\end{equation*}

where $\mathrm {T_{pulse}}$ is the total pulse duration of the stimulating waveform in milliseconds, $\mathrm {B_{norm}}$ represents the PNS threshold normalized by the rheobase (such that as $\mathrm {T_{pulse}\to \infty }$, $\mathrm {B_{norm}\to 1}$), and fit parameters $\alpha = 0.44, \beta = 4.32, \text {and}~~ \gamma =0.60$. We apply (9) to normalize our pulses from constant number of cycles to constant total pulse duration across all frequencies to eliminate the effect of total pulse duration on the PNS threshold.

## III. Results

Fig. 1 shows an image of the coil windings, an illustration of the coil around a head model, and field efficiency measurements overlaid with FEMM field simulations. We measure a $214~\mathrm {\mu T/A}$ field efficiency at the coil center, compared to a simulated $212~\mathrm {\mu T/A}$. The greatest error between the measured and simulated field maps occurs at the periphery of the measured field of view. Radially ( $\hat {\mathrm {r}}$) 100 mm from the coil center, we measure a $288~\mathrm {\mu T/A}$ field efficiency vs. a simulated $272~\mathrm {\mu T/A}$ field efficiency (Fig. 1c). Axially ( $\hat {\mathrm {z}}$) 60 mm from the coil center (towards the subject body), we measure a field efficiency of $175~\mathrm {\mu T/A}$ vs. a simulated $181~\mathrm {\mu T/A}$ (Fig. 1d).

**FIGURE 3.**
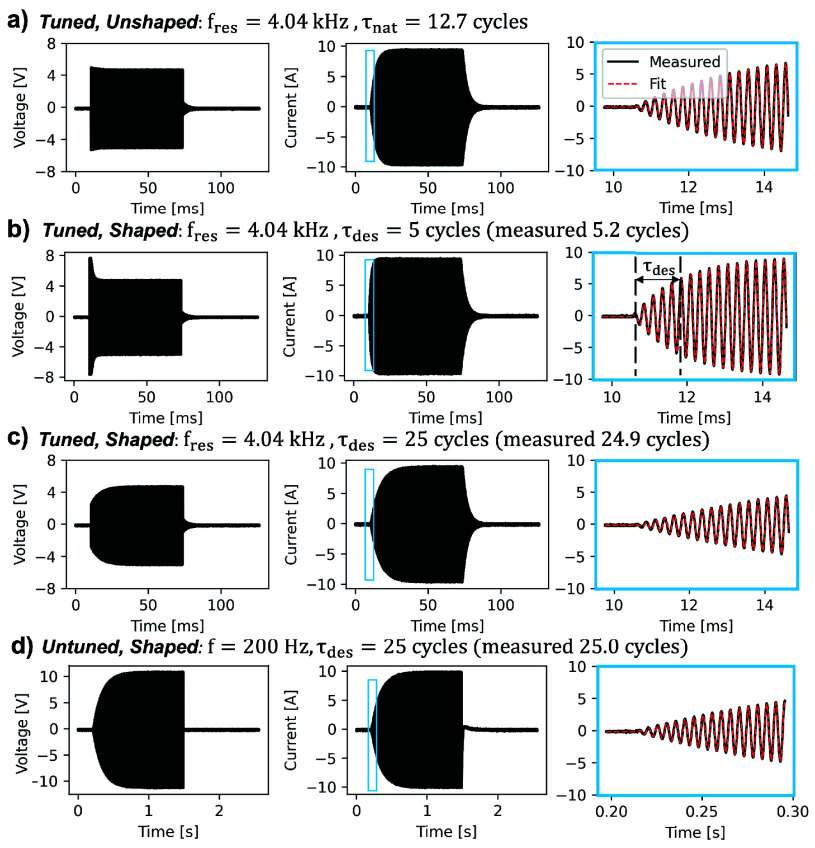
Pulse shaping examples. We shaped current pulses to eliminate differences in pulse rampup times to enforce similar waveform shapes across all frequencies. Each row shows the voltage waveform (left), current waveform (middle), and zoom in of current waveform to show fit function (equation (3)) superimposed in dashed red. (a) Measured 4.04 kHz step voltage input and current response, which has a natural time constant of 12.7 cycles. (b) Shaped 4.04 kHz voltage waveform to achieve a desired time constant of 5 cycles (measured as 5.2 cycles). $\mathrm {\tau _{des}}$ is marked during the rampup phase for this waveform. (c) Shaped 4.04 kHz voltage waveform to achieve a rampup time constant of 25 cycles (measured 24.9 cycles). (d) Example untuned 200 Hz waveform, modulated by an exponential envelope with 25 cycle rampup time constant (25.05 measured). In the experiments, a time constant of 25 cycles for all resonant and untuned frequencies was chosen.

Fig. 3a-c show the effect of pulse shaping on the voltage (left panels) and current (middle panels) waveforms for the $\mathrm {f} =4.04$ kHz resonant condition. The natural time constant associated with the rampup envelope of the 4.04 kHz current waveform is $\mathrm {\tau _{nat}} =12.7$ cycles/f. We modulated the voltage envelopes to achieve target time constants $\mathrm {\tau _{des}}$ of 5 and 25 cycles/f in the current waveforms in Fig. 3b and Fig. 3c respectively. The right panels show a zoom on the onset of the current waveforms of form (3), overlaid with a fit function from which we obtained the steady state current amplitude and extracted the measured ramp-up time constants to experimentally verify correct pulse shaping. Across all pulses, we achieved a pulse rampup distribution with a mean of 24.8 cycles and standard deviation of 0.91 cycles, indicating good performance of the pulse shaping targeting 25 cycles rampup. For comparison, Fig. 3d shows pulse shaping for the 200 Hz untuned condition. Note that in the untuned case, there is no ringdown in the current waveform like in the tuned cases.

**FIGURE 4.**
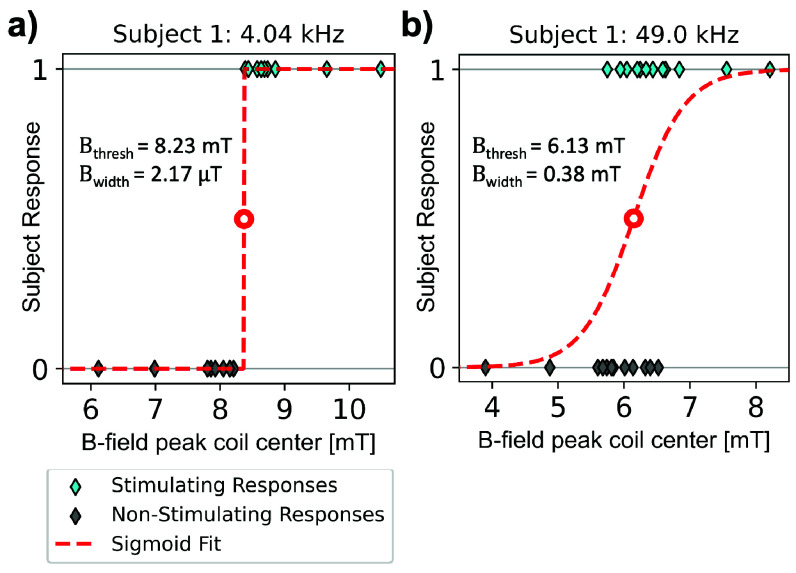
Example subject responses for threshold determination. Subjects record stimulation via a push button for each pulse over peak amplitude at the coil center. (a) Example of a clear transition between no stim and stim, where there is no overlap in subject response verses field amplitude. (b) Example of a wide transition between no stim and stim with some ambiguity.

Fig. 4 plots subject responses (stimulation or no stimulation) as a function of peak B-field amplitude at coil center, superimposed with sigmoid fits in (5). In Fig. 4a, there is a sharp transition between non-stimulating and stimulating B-field amplitudes for a 4.04 kHz pulse. The fitted threshold in this case is $\mathrm {B_{th}} =8.23$ mT with a sigmoid width of $\mathrm {B_{width}} = 2.17~\mathrm {\mu T}$. In Fig. 4b, there is some overlap between the non-stimulating and stimulating amplitudes for a 49.0 kHz pulse. This results in a broader sigmoid width ( $\mathrm {B_{th}} =6.13$ mT, $\mathrm {B_{width}} =0.38$ mT).

**FIGURE 5.**
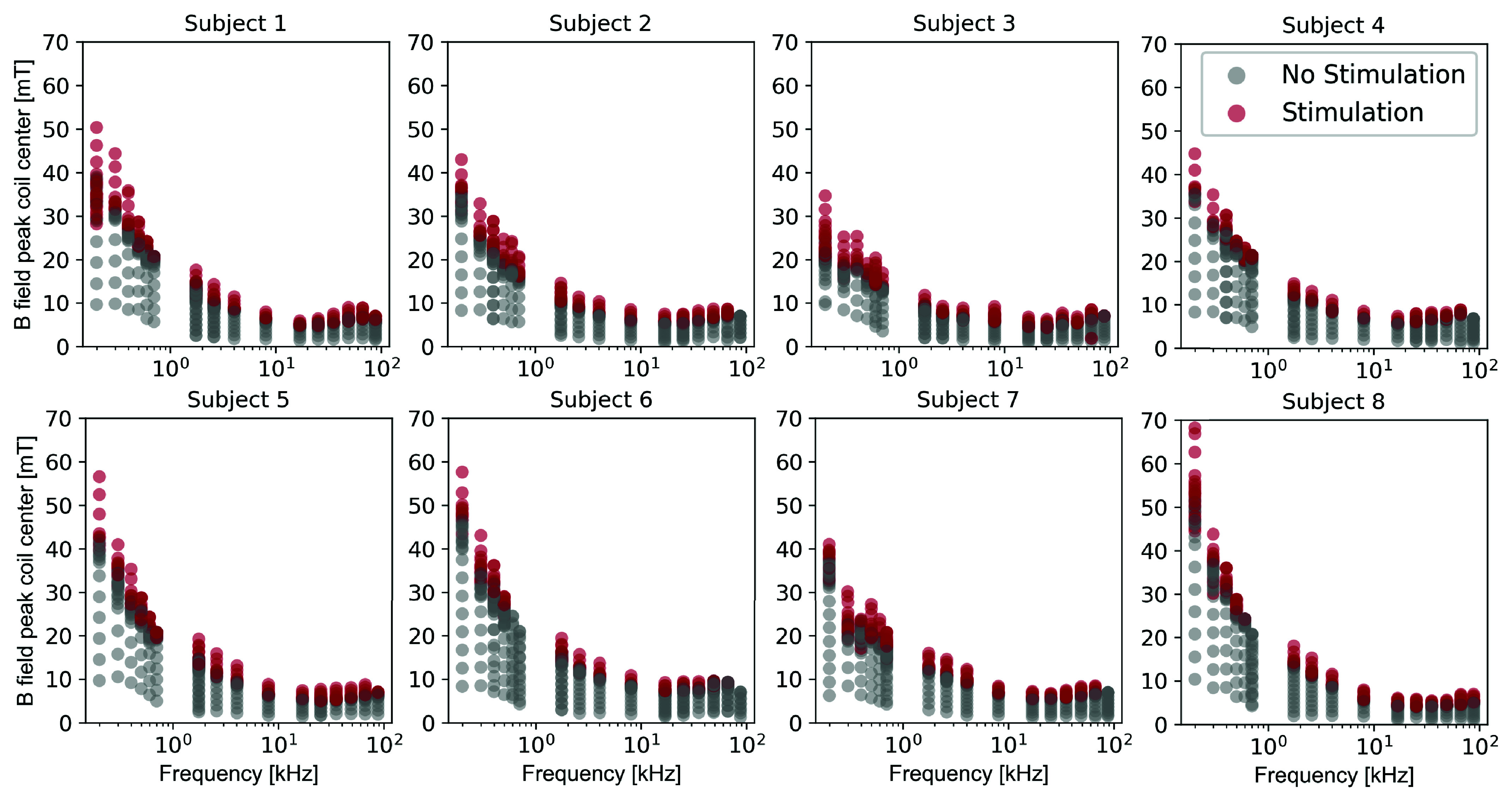
All subject titration data versus frequency on a log scale. Field amplitudes represent the peak B-field at the coil center. Each dot represents an applied B-field pulse. Red dots represent subject reported stimulation at the B-field frequency and amplitude. Grey dots represent no subject reported stimulation. Each vertical set of dots represents a single titration run for a particular frequency.

Fig. 5 shows subject responses measured in each of the eight subjects at different B-field amplitudes at coil center (y-axes) for all frequencies (x-axes). Red points denote a subject reporting stimulation at a given field amplitude, and gray points indicate no stimulation. All stimulation sites reported by the subjects during the experiments are presented in Supplementary Fig. S2.

Table 1 presents the 1.76 kHz and 25.3 kHz test-retest PNS thresholds in units of B-field at coil center, and widths of the sigmoid fits in mT measured in five subjects. Supplementary Fig. S3 shows Bland-Altman plots for the two frequencies. The largest threshold difference observed for the 1.76 kHz test-retest was a 10.0% increase for subject 6 from 13.9 mT to 15.3 mT, and a threshold decrease of 7.7% for subject 4 from 6.14 mT to 5.67 mT for the 25.3 kHz test-retest.

**TABLE 1:** Test-Retest PNS Thresholds and Widths Obtained From Sigmoid Fits for 1.8 kHz and 25.3 kHz for Subjects 2-6

	1.8 kHz				25.3 kHz			
Subject	Measurement 1		Measurement 2		Measurement 1		Measurement 2	
	$\mathrm {B}_{\mathrm {th}}$ [mT]	$\mathrm {B}_{\text {wifth}~}$ —mT—	$\mathrm {B}_{\mathrm {ta}}$ [mT]	$\mathrm {B}_{\text {with}~}$ [mT]	$\mathrm {B}_{\mathrm {th}}$ [mT]	$\mathrm {B}_{\text {width}~}$ [mT]	$\mathrm {B}_{\mathrm {th}}$ [mT]	$\mathrm {B}_{\text {width}~}$ [mT]
2	10.21	0	10.09	0.01	5.21	0	5.35	0.13
3	9.06	0.17	8.56	0.42	4.6	0.07	4.43	0.26
4	11.9	0.32	11.94	0	6.08	0.11	5.61	0.01
5	12.9	0.97	13.77	0.54	4.83	0.09	4.86	0
6	13.8	0.25	15.19	0.29	7.6	0.21	7.42	0.21

**FIGURE 6.**
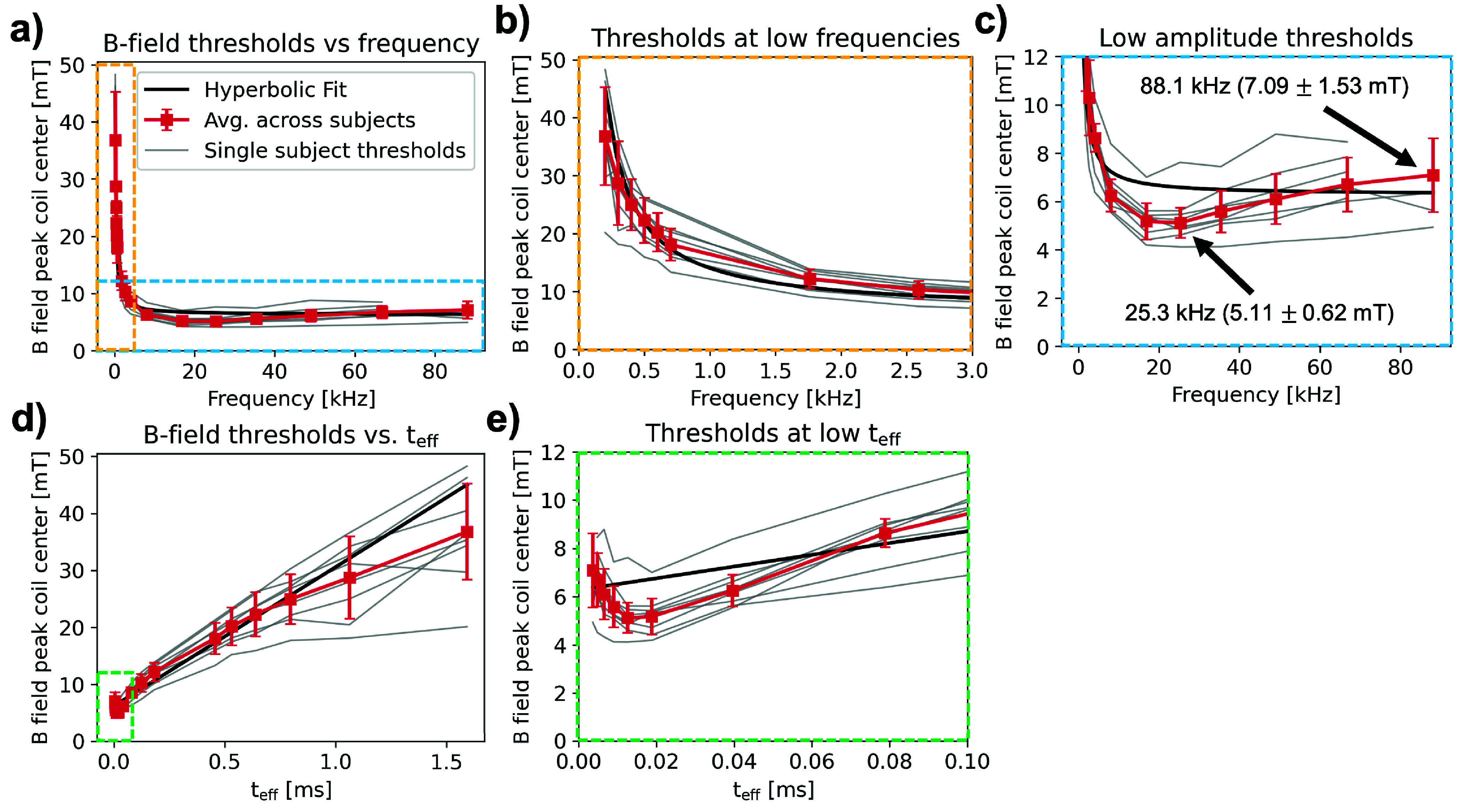
(a) All subject threshold data on linear frequency scale. Gray traces represent a single subject’s threshold vs. frequency curve. Overlaid are averages across subjects calculated by fitting error functions to a normal CDF (red trace). Error bars indicate standard deviation of the fit CDF. The black curve is a hyperbolic strength-duration curve fit of the subject-averaged thresholds ( $\mathrm {B_{rheo}} =6.27$ mT, $\mathrm {\tau _{chron}} = 405~\mathrm {\mu }$s). Note that the hyperbolic fit was performed using frequency points below 10 kHz but extrapolated at all frequencies. (b) Zoom into lower frequency data points on linear scale. (c) Zoom into lower amplitude threshold region, to emphasize deviation from hyperbolic SDC for high frequency data points. (d) All subject data plotted against effective stimulus duration, $\mathrm {t_{eff}}$, where $\mathrm {t_{eff} = 1 / (\pi f)}$ for a sinusoidal stimulation waveform. (e) Zoom into lowest $\mathrm {t_{eff}}$ (highest frequency) points to highlight deviation from the hyperbolic fit.

Fig. 6a shows measured PNS thresholds in units of peak B-field at coil center plotted against frequency on a linear scale. Gray curves show threshold measurements for single subjects, and the red curve shows the average measured threshold (red squares) and standard deviation (error bars) across all subjects determined from a CDF fit to individual PNS thresholds for each frequency using (6). We found that the measured thresholds varied between 9.14% (700 Hz) and 23.2% (200 Hz) (standard deviation over average thresholds) between subjects. The maximum threshold relative to mean was 1.44 (25.3 kHz) and the minimum threshold relative to mean was 0.64 (300 Hz). Supplementary Fig. S4 shows individual CDF fits computed for each frequency. We also fit a hyperbolic SDC in (7) (black curve) to the mean PNS thresholds for frequencies $\leq 10$ kHz to compare the measured thresholds with trends predicted by the hyperbolic SDC. The fitted rheobase is $\mathrm {B_{rheo}} =6.27$ mT, and the fitted chronaxie is $\mathrm {\tau _{chron}} = 405~\mathrm {\mu s}$.

The yellow and blue boxes in Fig. 6a show an x-axis zoom into the low frequency portion of the curve (Fig. 6b) and a y-axis zoom into the lower threshold amplitudes (Fig. 6c), respectively. For each subject, the measured PNS threshold curve shows a characteristic minimum between 16.9 kHz and 25.3 kHz, followed by increasing thresholds at higher frequencies (Fig. 6c). Specifically, the average threshold at 88.1 kHz is 7.09 mT $\pm ~1.53$ mT (mean ± standard deviation), which is 38.7% higher than the average threshold measured at 25.3 kHz (5.11 mT $\pm ~0.62$ mT). The observed average PNS threshold increase between 25.3 kHz and frequencies $\geq 49.0$ kHz is statistically significant at the p =0.05 significance level (independent samples t-test). The increase in measured threshold at high frequencies deviates from the hyperbolic SDC, which predicts an asymptotic threshold decrease towards the rheobase, $\mathrm {B_{rheo}}$.

Fig. 6d shows the same data as in Fig. 6a-c plotted against effective pulse duration $\mathrm {t_{eff}}$ (8). Note that the hyperbolic fit versus frequency becomes linear when plotted against $\mathrm {t_{eff}}$. Fig. 6e shows thresholds plotted at low $\mathrm {t_{eff} \leq }~0.10$ ms ( $\mathrm {f \geq }~4.0$ kHz) points for better visibility (green box in Fig. 6d). These plots again show deviations of the measured PNS thresholds from the hyperbolic SDC, especially at low stimulus durations (high frequencies).

**FIGURE 7.**
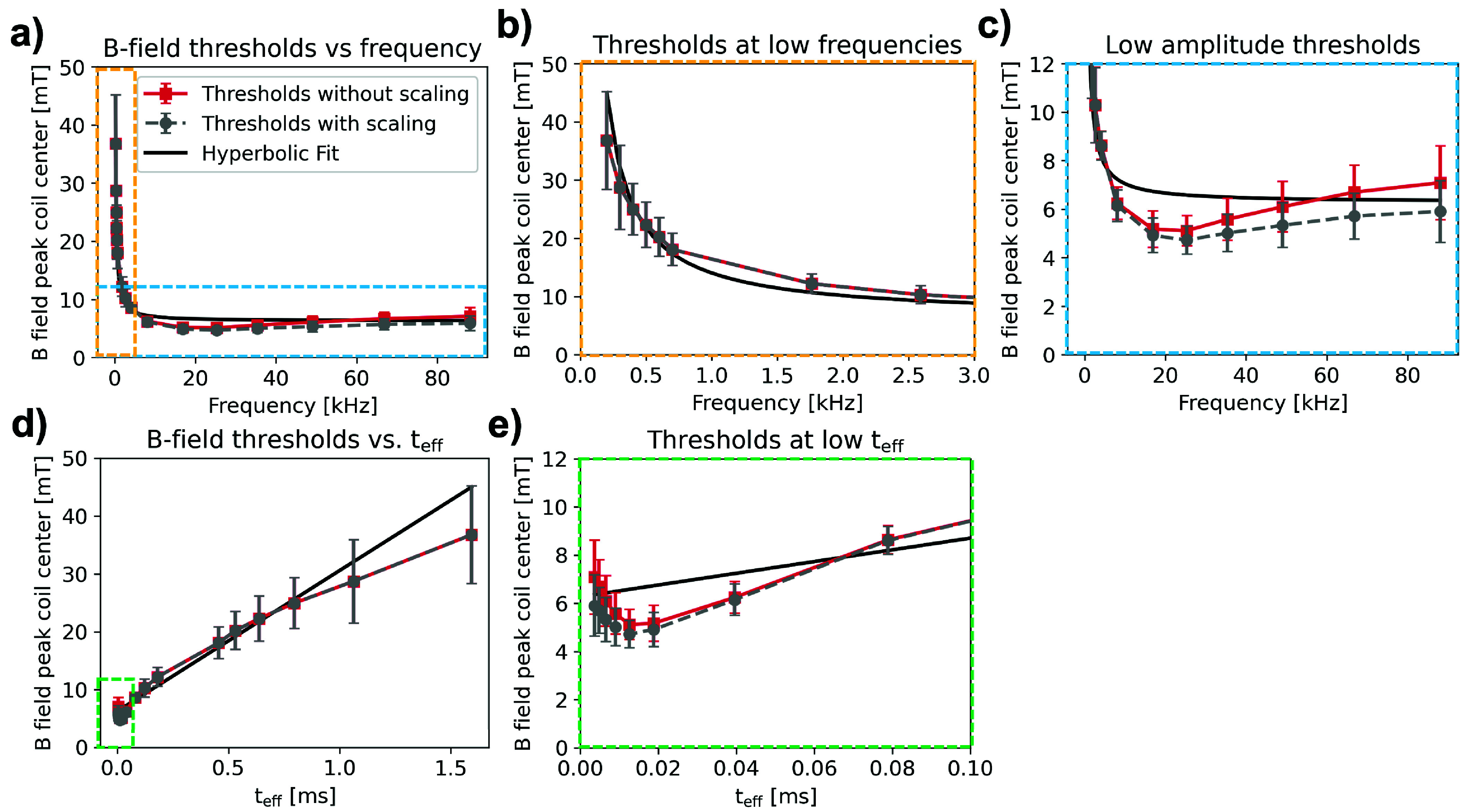
(a) Comparison of all subject averages (red trace) for constant 256 cycles per sinusoidal pulse, with application of equation (8) to scale thresholds to constant pulse duration (gray trace). Overlaid is the hyperbolic fit from Fig. 6 (black trace). (b) Zoom into lower frequency data points. Note that the duration scaling does not alter thresholds significantly over these frequencies. (c) Zoom into lower amplitude threshold region. Because of the short pulse durations at these frequencies, we see up to 17% lower thresholds at 88.1 kHz in the corrected trace compared to the uncorrected trace. (d) The same data from (a), plotted versus $\mathrm {t_{eff}}$. (e) Zoom into lowest range of $\mathrm {t_{eff}}$.

In this work, we applied stimulating pulses with a constant number of 256 cycles per pulse for each frequency. We used the scaling law developed by Saritas et al. [26] (9) to adjust our results to constant pulse duration for easier comparison to previous PNS measurements. Fig. 7 shows the average PNS thresholds in red and the hyperbolic fit curve in black (same data as shown in Fig. 6). The gray curve shows average thresholds scaled to infinite pulse duration, i.e., to the threshold rheobase, using (9). When scaling to infinite pulse duration, we see negligible threshold changes for frequencies <10 kHz, and decreased thresholds for frequencies $\mathrm {\geq }~10$ kHz. The 25.3 kHz threshold scaled to infinite duration (4.72 mT $\pm ~0.58$ mT) is 25% lower than the average scaled 88.1 kHz threshold (5.91 mT $\pm ~1.27$ mT) and is not statistically significant (p =0.053, independent samples t-test). However, the observed increase between the scaled 25.3 kHz threshold and the scaled 66.7 kHz threshold ( $5.71~\pm ~0.95$ mT) is statistically significant at the p =0.05 significance level (p =0.025).

## IV. Discussion and Conclusion

In this study, we measured peripheral nerve magnetostimulation thresholds in the heads of eight healthy volunteers using a solenoidal drive coil intended for human head MPI at 16 frequencies between 200 Hz and 88.1 kHz. Both measured PNS thresholds in individual volunteers and average thresholds across volunteers deviate from the hyperbolic SDC’s asymptotic behavior for frequencies greater than 25 kHz (Fig. 6c). Specifically, we found a significant (p =0.011, t-test) 39% average PNS threshold increase from 5.11 mT $\pm ~0.62$ mT at 25 kHz to 7.09 mT $\pm ~1.53$ mT at 88.1 kHz. Our data shows conclusively that magnetostimulation thresholds reach a minimum value at 25 kHz. This contrasts the hyperbolic SDC, which predicts hyperbolic monotonically decreasing thresholds with increasing frequency. Thus, the hyperbolic SDC does not capture the behavior of PNS at frequencies above 25 kHz. Understanding magnetostimulation in this high frequency regime is critical for optimizing therapeutic neuromodulation applications such as treatment of chronic pain (nerve block [27] or spinal cord stimulation [28]) or to assess the safety limits of human MPI scanners [19], [21], [22], [29] and high-frequency MRI gradient systems [15].

The hyperbolic SDC is often used to describe the frequency behavior of PNS thresholds and uses only two parameters, the threshold rheobase $\mathrm {B_{rheo}}$ and the chronaxie $\mathrm {\tau _{chron}}$. We have previously demonstrated that the chronaxie is influenced by coil position, local anatomical features, and by intrinsic nerve axon properties [30] (e.g., the nerve bend angle and radius), which may explain the wide range of chronaxie values observed in simulations and experiments (3-4 fold in the head [8], [31]). Furthermore, the quantitative PNS threshold (and $\mathrm {B_{rheo}}$) is known to be affected by a multitude of factors such as coil geometry, stimulation waveform, subject position relative to coil windings, as well as nerve type, diameter, and orientation relative to the induced E-field [6], [30], [32]. None of these factors are captured by the hyperbolic SCD, and there is no set of universal chronaxie and rheobase values that is applicable to all coils, waveforms, or stimulated body parts [30].

Our measurements here reveal further shortcomings of the hyperbolic SDC, namely its failure to capture the experimentally observed increase in thresholds above 25 kHz. Even at low frequencies (<1 kHz), the hyperbolic SDC does not perfectly describe our measured PNS thresholds. This can best be seen in the linearized plot in Fig. 6d, which shows measured B-field thresholds as a function of effective stimulus duration. In this regime, the SDC becomes linear and deviations from the measured thresholds at both low frequencies (long stimulus durations) and high frequencies (short stimulus durations) become more apparent. We therefore argue that while the SDC might be suitable to qualitatively describe threshold curves of single nerve stimulation experiments in simple setups and within limited frequency ranges, it may not be suitable to predict PNS thresholds in more realistic in-vivo setups and at wider frequency ranges. Despite these limitations, the deviations between measured PNS thresholds and the hyperbolic SDC model are modest within the MRI operating frequency range (0-2 kHz, Fig. 6b, or $\mathrm {t_{eff}> 0.16}$ ms, Fig. 6d). To be clear, we do not argue in this paper for a change of PNS model for safety assessment in MRI (IEC 60601-2-33).

The hyperbolic SDC is based on a neuronal model with constant excitability over the frequency range and linearly increasing electric field strength with frequency (Faraday induction). Clearly, the first assumption is not correct over the extended frequency range studied in this work. Specifically, the hyperbolic SDC does not match the increase of thresholds above 25 kHz. This could be due to the limited time constant of sodium ion channels (1-10 ms [33]) that limit transmembrane ion dynamics following fast external potential changes, thus preventing the initiation of action potentials. Our group has also shown in simulations and experiments that application of a 10 kHz stimulus can increase potassium permeability, which can raise the threshold for action potential generation in peripheral nerves [34]. Another interesting effect described by Schmale et al. is capacitive shorting of the nerve membrane due to increased cell conductivity at high frequencies (beta dispersion) [17], [35], [36]. Many, but not all, of those effects, are included in the state-of-the-art physiological model of myelinated nerves, namely the McIntyre-Richardson-Grill (MRG) model [37]. Therefore, more experimental and numerical studies of PNS at high frequencies are needed to fully understand the observed threshold-frequency behavior.

The intra-subject repeatability of our measurements was within approximately 5% except for subject 4, who exhibited a 10% decrease in threshold at 25.3 kHz between test-retest measurements. We were able to achieve this high level of robustness in threshold measurements by carefully coaching subjects in a training session prior to running the titrations. Subjects were instructed to minimize head motion to maintain head position relative to the coil through the duration of the experiment. If subjects exited the coil during a break session halfway through the experiment, we carefully repositioned their head following a standard protocol for the remainder of the session. Furthermore, the careful titration scheme to accurately determine the PNS threshold for each frequency aided in reducing variability of our measurements. Overall, we thus have high confidence in the robustness of our results.

Our study employed damped sinusoidal pulses resulting from the natural oscillations of LCR circuits. Translation of our sinusoidal threshold results to trapezoidal waveforms more commonly used in MRI is facilitated by our use of $\mathrm {t_{eff}}$. This is a measure of gradient rise defined in IEC 60601-2-33 applicable to arbitrary waveforms. The IEC defines $\mathrm {t_{eff}}$ as the difference between the amplitudes of the first two waveform peaks divided by the maximum derivative of the waveform over this interval [5]. Fig. S5 in the Supplementary Information shows PNS thresholds for sinusoidal and trapezoidal waveforms from a previous publication by our group [6], plotted as a function of both the pulse duration (i.e., twice the rise time for the trapezoidal shape; half-period for the sinusoidal shape) and $\mathrm {t_{eff}}$. These show that $\mathrm {t_{eff}}$ is indeed useful to uniformize PNS thresholds across waveform types. However, small differences can remain between the thresholds associated with different waveforms despite using $\mathrm {t_{eff}}$. This is expected given the complexity of ion channel dynamics that are cannot be entirely captured by such a simple metric. A more accurate conversion of our results to trapezoidal waveforms may be obtained using simulations based on the MRG neurophysiological model of myelinated nerves [37], however it is not clear whether such an approach would yield accurate results above 10 kHz where the accuracy of MRG is unknown. Most likely, an accurate characterization of thresholds for trapezoidal waveforms would require direct measurements, but those are much more difficult to obtain experimentally than the sinusoidal waveforms used here.

In our study, we employed stimulating pulses with a constant number of 256 bipolar cycles across all frequencies, which led to total waveform durations of 1280, 853, 640, 512, 427, 366, 146, 98.8, 63.4, 31.8, 15.1, 10.1, 7.23, 5.22, 3.84, 2.91 ms at 0.2, 0.3, 0.4, 0.5, 0.6, 0.7, 1.75, 2.59, 4.04, 8.05, 16.9, 25.3, 35.4, 49.0, 66.7, 88.1 kHz respectively. Such pulses are typically used in PNS experiments conducted to assess the PNS limit of novel MR gradient coils. Those experiments typically use waveforms with a constant number of cycles or lobes, while varying other parameters such as rise time (i.e., the time it takes to ramp up the gradient field from zero to peak), or flat top duration for trapezoidal gradient pulses [4], [8], [9], [11], [12], [31]. Budinger et al. observed decreasing PNS thresholds with increasing number of cycles in their stimulating waveforms [38], approaching an asymptotic value for waveforms with 16 cycles or more. However, their measurements were limited to a single frequency of 1.27 kHz. Saritas et al. expanded upon Budinger et al.’s work, conducting PNS experiments in the forearm at 1.2, 5.7, and 25.5 kHz, while varying total pulse duration from 2 ms to 125 ms for each frequency [26]. They found that PNS thresholds approach a minimum asymptote for overall pulse duration of >20ms for all frequencies, rather than for pulses with a fixed number of cycles. They also developed a model that describes the relationship between rheobase-normalized PNS thresholds and total pulse duration. This model could facilitate comparison of PNS measurements across different sinusoidal pulse durations, assuming this model holds across body parts, waveform shapes, and beyond the frequency range studied by Saritas et al. In MPI, drive fields are played as continuous waves, i.e., for a very large number of pulses (~infinite duration). We adjusted our measurements to infinite duration using the scaling relation by Saritas et al. [26] to relate our PNS measurements to waveforms that are more realistic for MPI. Our pulses at frequencies $\geq 8.05$ kHz had total durations of $\leq 20$ ms, which is shorter than the asymptote identified by Saritas’ scaling relation. Consequently, the thresholds measured at $\geq 8.05$ kHz decrease after applying Saritas’ relation, with a more pronounced threshold reduction at higher frequencies. The observed PNS threshold increase above 25 kHz however, remained statistically significant.

It is known that both the shape of the waveform and the total number of cycles influence PNS thresholds. Indeed our initial tests suggested an effect of the number of cycles in the ramp-up period of the pulse of 5-10%. This effect is a confounding factor in this study, which focuses on the effect of frequency on thresholds, not pulse shape. Therefore all the pulses in this study use a constant number of cycles in the ramp-up envelope, and a constant number of total cycles.

Ozaslan et al. measured PNS in the human head at 24 kHz, which is a typical MPI frequency [19]. They observed a $3.61~\pm ~0.82$ mT average threshold in three subjects for 50 ms pulses at 24 kHz pulses. In comparison, we measured an average threshold of $5.11~\pm ~0.62$ mT at coil center at 25.3 kHz (256 cycles, corresponding to a total pulse duration of 10.1 ms). Applying Sarita’s scaling relation to our measurements yields an average threshold of $4.72~\pm ~0.58$ mT for a 25.3 kHz pulse with 50 ms duration, which is ~30% higher than Ozaslan et al.’s measurement. This discrepancy is likely due to the differing coil geometries, subject position and anatomies, and waveform parameters. Both Ozaslan et al. and our findings indicate that PNS can potentially become a limiting factor for human head MPI systems, which typically operate at B-field amplitudes up to 5 mT at coil center and at frequencies around 25 kHz. This is unfortunate, as our data shows that PNS thresholds are lowest near that frequency. Specifically, PNS can limit MPI sensitivity by reducing the usable drive field amplitude, which may be insufficient to drive the super paramagnetic oxide nanoparticle (SPION) tracers used for MPI signal generation. Possible avenues to mitigate PNS restrictions for MPI include tailoring SPION properties to field amplitudes <5 mT or designing PNS-optimized coils with inherently higher PNS thresholds, which our group has done successfully for MRI gradient coils [39].

The main objective of this study was to characterize magnetostimulation PNS thresholds across a wide range of frequencies in the healthy population. Using 8 subjects, we were able to draw statistically significant conclusions about the variation of thresholds with frequency. However, this sample size is too small to draw conclusions about the variation of thresholds across body habitus, gender or age [40]. For example, we did not observe significant correlations between thresholds and subject weight, size, head circumference or age. Current safety guidelines for MRI and MPI rely on population-average PNS thresholds. While it is not clear whether patient-specific PNS characterization could improve the safety or performance of these imaging modalities, this question remains open but beyond the scope of this work. Thus, we may explore it in future studies.

In summary, our measurements demonstrate that the hyperbolic SDC is not applicable for sinusoidal stimulation waveforms at frequencies above 25 kHz, a consistent finding with trends observed in previous studies. Further studies are needed to uncover the mechanisms of action of PNS at frequencies beyond 25 kHz, which is critical for the development of therapeutic nerve stimulation applications and for setting appropriate safety limits for the use of MRI gradient and MPI systems.

## Data Availability Statement

The authors make all data and materials supporting the results or analyses presented in their paper available at https://github.com/acbarksdale/high_frequency_head_pns_ threshold_dataset

## Supplementary Materials

Supplementary Materials
