# Supplementary material for: Measurement of Peripheral Nerve Magnetostimulation Thresholds of a Head Solenoid Coil Between 200 Hz and 88.1 kHz
Source: IEEE J Transl Eng Health Med. 2025 May 15;13:275–85. doi: 10.1109/JTEHM.2025.3570611 (PMC12310169; doi:10.1109/JTEHM.2025.3570611)
Supplement: Supplementary Materials [file supp1-3570611.pdf]

# Measurement of peripheral nerve magnetostimulation thresholds of a head solenoid coil between 200 Hz and 88.1 kHz

**Alex C. Barksdale[1,2,*], Natalie G. Ferris[2,3,4], Eli Mattingly[2,4], Monika ´Sliwiak[2], Bastien Guerin[2,5], Lawrence L. Wald[2,4,5], Mathias Davids[2,5], Valerie Klein[2,5]**

1 MIT Department of Electrical Engineering and Computer Science, Cambridge, MA, United States
2 Martinos Center for Biomedical Imaging, Charlestown, MA, United States
3 Harvard Graduate Program in Biophysics, Harvard University, Cambridge, MA, United States
4 Harvard-MIT Division of Health Sciences and Technology, Boston, MA, United States
5 Harvard Medical School, Boston, MA, United States
* Corresponding author: Alex C. Barksdale, email: abarksdale@mgh.harvard.edu

## Supplementary Information

**Table S1**: Typical measured characteristics of tuned waveforms conditions prior to pulse shaping. The untuned characteristics are used to determine the voltage envelope for shaping the current waveform. The resonant frequencies, rampup time constants in milliseconds and number of cycles for each resonant frequency, in addition to the series resistance at resonance are presented. Resonant frequency and rampup time constants are obtained by fitting $I(t) = I_0(1 - \exp(-t/\tau))\sin(2\pi f_{res} t + \phi)$ over $I_0$, $\tau$, $f_{res}$, and $\phi$. $R_{series}$ is obtained by additionally fitting $V(t) = V_0 \sin(2\pi f_{res} t + \phi)$ to the applied voltage waveforms, and computing $R_{series} = V_0/I_0$ assuming real impedance at resonance.

| $f_{res}$ [kHz] | $\tau$ [ms] | $\tau$ [cycles] | $R_{series}$ [Ω] |
|---|---|---|---|
| 1.75 | 4.65 | 8.12 | 0.256 |
| 2.59 | 3.64 | 9.43 | 0.373 |
| 4.04 | 3.15 | 12.7 | 0.527 |
| 8.05 | 1.80 | 14.5 | 0.752 |
| 16.9 | 1.25 | 21.2 | 1.23 |
| 25.3 | 0.871 | 22.0 | 1.70 |
| 35.4 | 0.631 | 22.3 | 2.33 |
| 49.0 | 0.452 | 22.2 | 3.29 |
| 66.7 | 0.314 | 21.0 | 4.62 |
| 88.1 | 0.216 | 19.1 | 6.55 |

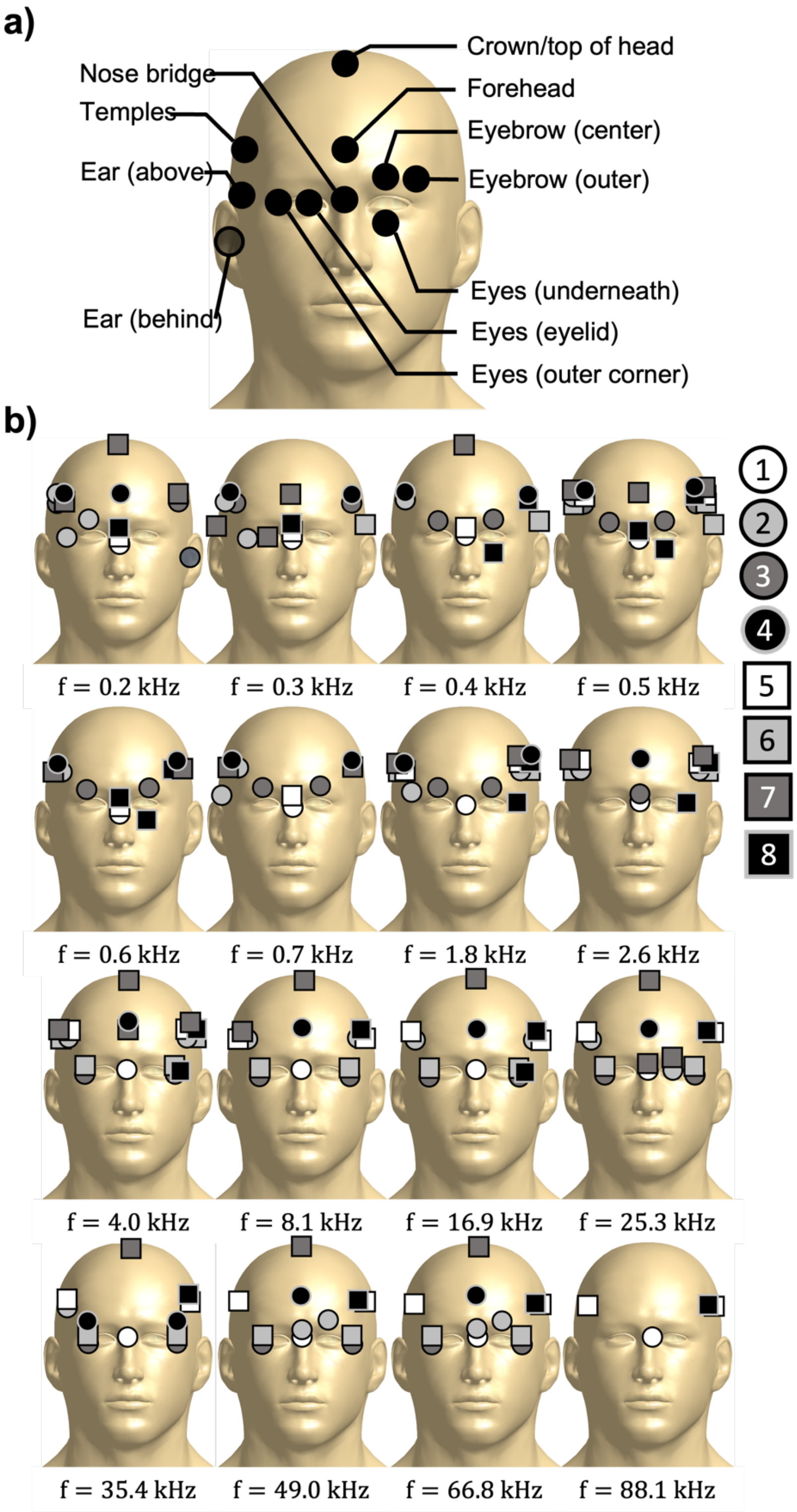


**Figure S2**: **(a)** Sites reported by subjects during PNS experiments mapped to generic head model. Black dots represent a site reported by any subject across all frequencies at any point during a titration. **(b)** Distribution of stimulation sites across subjects for each frequency condition. In some cases, subjects did not report stimulation, and the corresponding subject marker is absent from the head model.

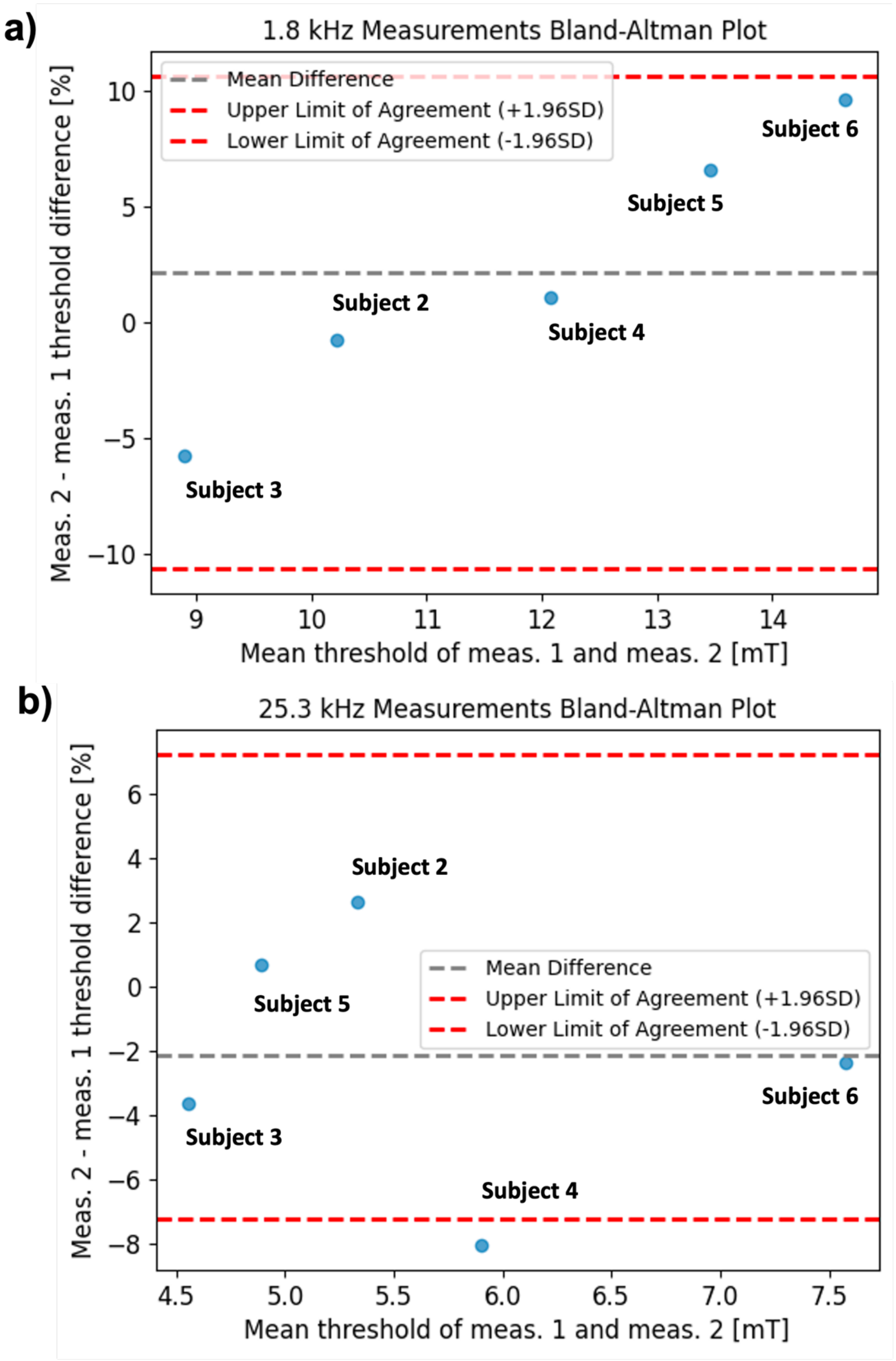


**Figure S3**: (**a)** Bland-Altman plot of PNS threshold pairs measured in 5 subjects at 1.8 kHz. (**b)** Bland-Altman plot of PNS threshold pairs measured in 5 subjects at 25.3 kHz.

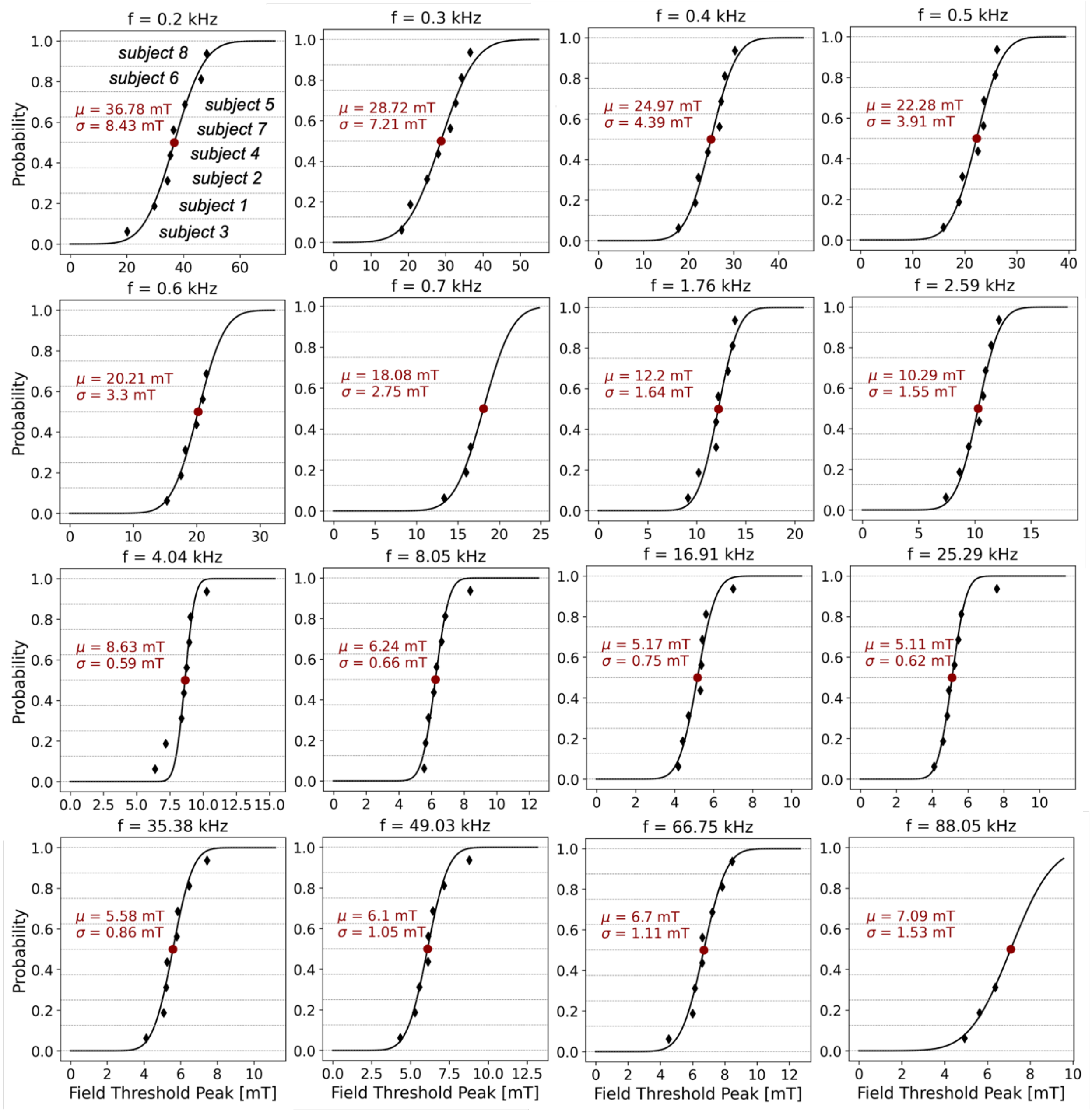


**Figure S4:** Error function estimation of the mean PNS threshold across subjects for each frequency. Each point presents the threshold measured for a single subject. In the top-left most pane, each subject data point is labeled with subject number to demonstrate construction of the CDF for each frequency (ordering changes across panes based on the field threshold peak for each frequency). For some subjects and frequencies, the PNS threshold could not be reached within the amplifier limits. We constructed CDFs assuming normally distributed thresholds across subjects and fitted the corresponding mean and standard deviation to impute missing data points. Note that for 88.1 kHz, only 3 out of 8 subjects reported stimulation, resulting in a relatively large standard deviation about the estimated mean (mu=7.09 mT, sigma=1.53 mT).

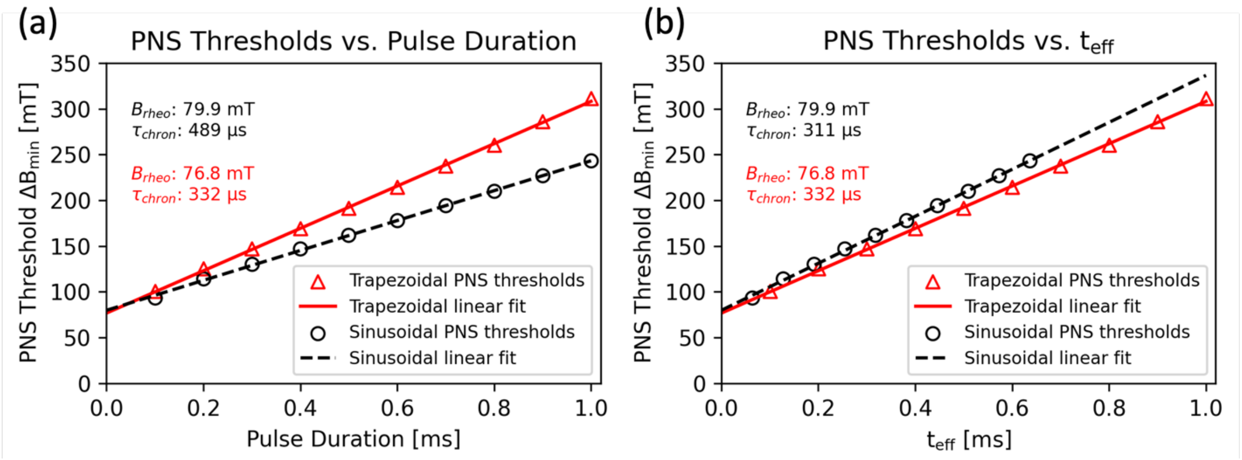


**Figure S5:** PNS thresholds as a function of (a) pulse duration, and (b) the IEC-defined $t_{eff}$ (60601-2-33). For a trapezoidal waveform, $t_{eff}$ is equal to the pulse duration. For a sinusoidal waveform, $t_{eff}$ = $(2/\pi)$ * T/2, where T is the sine period. Using teff as a measure of gradient rise time allows some level of uniformization of PNS thresholds across waveform types, although some differences remain. Data is from a previous publication by our group [1].

[1] Davids, Mathias, et al. "Predicting magnetostimulation thresholds in the peripheral nervous system using realistic body models." Scientific reports 7.1 (2017): 5316.

## Pulse Shaping

### A. LCR Second Order System Analysis

In the resonant coil case, the series LCR circuit is modeled as a second order system. The dynamics of the LCR circuit can be expressed as:

$$v(t) = L\frac{di(t)}{dt} + \frac{1}{C}\int_{-\infty}^{t} i(t')dt' + Ri(t)$$

Taking the derivative with respect to t, and dividing through by L:

$$\frac{1}{L}\frac{dv(t)}{dt} = \frac{d^2i(t)}{dt^2} + \frac{R}{L}\frac{di(t)}{dt} + \frac{1}{LC}i(t)$$

Taking the Laplace transform of the above equation, assuming all zero initial conditions yields:

$$\frac{1}{L}sV(s) = s^2I(s) + \frac{R}{L}sI(s) + \frac{1}{LC}I(s)$$

Rearranging the above, and substituting $\alpha = R/2L$, and $\omega_0 = 1/\sqrt{LC}$, the circuit's voltage to current impulse response can be expressed as

$$H_{nat}(s) = \frac{I(s)}{V(s)} = \frac{1}{L}\frac{s}{s^2 + 2\alpha s + \omega_0^2}$$

where the subscript "nat" refers to the natural impulse response to a step input into the circuit. To determine the response to a sinusoidal step voltage input such as $v_{src}(t) = V_0 \sin(\omega t)\,\theta(t)$, we compute the Laplace transform of the time domain signal:

$$V_{src}(s) = \mathcal{L}\{V_0 \sin(\omega_0 t)\,\theta(t)\} = V_0 \frac{\omega_0}{s^2 + \omega_0^2}$$

and multiply by the natural impulse response above. Here, $\theta(t)$ denotes the Heaviside step function. Taking the inverse Laplace transform of this result:

$$I_{nat}(s) = V_{src}(s)H_{nat}(s) = \frac{V_0}{L}\frac{\omega_0}{s^2 + \omega_0^2}\frac{s}{s^2 + 2\alpha s + \omega_0^2}$$

$$i_{nat}(t) = \mathcal{L}^{-1}\{I_{nat}(s)\} = \frac{V_0}{2\alpha L}\left(\sin(\omega t) - \frac{\omega_0}{\sqrt{-\alpha^2 + \omega_0^2}}\sin\left(\sqrt{-\alpha^2 + \omega_0^2}\, t\right)e^{-\alpha t}\right)\theta(t)$$

In the limiting case where $\alpha \ll \omega_0$, the radical terms approximately simplify as $\sqrt{-\alpha^2 + \omega_0^2} \approx \omega_0$. After substitution of $\alpha = R/2L$ then the result above simplifies to:

$$i_{nat}(t) \approx \frac{V_0}{2\alpha L}(1 - e^{-\alpha t})\sin(\omega_0 t)\theta(t)$$

$$= \frac{V}{R}(1 - e^{-\alpha t})\sin(\omega_0 t)\,\theta(t)$$

where we observe that the input sinusoid is modulated by an exponentially decaying envelope function with time constant determined by $\alpha$, and the steady state amplitude is the applied voltage signal amplitude divided by the resistance of the LCR circuit. There is no phase shift associated with the current waveform relative to the voltage waveform. From an intuitive point of view, operating the LCR circuit at resonance results in cancellation of the imaginary impedances presented by the inductor and capacitor. This results in only the real resistance of the circuit apparent to the amplifier, accounting for no phase in the current waveform relative to the voltage waveform, and further attenuation (amplification) of the voltage amplitude by the resistance value. The exponential envelope relates to the time required to ramp up the energy stored in the electric and magnetic fields in the capacitor and inductor respectively to the full oscillation amplitude.

### B. Tuned (Resonant) Pulse Shaping

Suppose we desire a current response of the form

$$i_{des}(t) = \frac{V_0}{2\alpha L}\left(\sin(\omega t) - \frac{\omega_0}{\sqrt{-\gamma^2 + \omega_0^2}}\sin\left(\sqrt{-\gamma^2 + \omega_0^2}\, t\right)e^{-\gamma t}\right)\theta(t)$$

$$=\frac{\gamma}{\alpha}\frac{V_0}{2\gamma L}\left(\sin(\omega t)-\frac{\omega_0}{\sqrt{-\gamma^2+\omega_0^2}}\sin\left(\sqrt{-\gamma^2+\omega_0^2}\,t\right)e^{-\gamma t}\right)\theta(t)$$

which has the steady state behavior of the natural response (as $t\rightarrow\infty$, the magnitude of the sinusoid approaches $V_0/2\alpha L = V_0/R$) but has the envelope characteristics governed by the time constant related to $\gamma$ rather than $\alpha$. This can be seen by again assuming that $\gamma \ll \omega_0$, such that $\sqrt{-\gamma^2+\omega_0^2}\approx\omega_0$. After applying this approximation, the desired current takes the form

$$i_{des}(t)\approx\frac{V_0}{R}(1-e^{-\gamma t})\sin\left(\omega_0 t\right)\theta(t)$$

Note that this system has impulse response given by:

$$H_{des}(s)=\frac{\gamma}{\alpha}\frac{1}{L}\frac{s}{s^2+2\gamma s+\omega_0^2}$$

where the prefactor $\gamma/\alpha$ accounts for the difference in the steady state amplitude of the waveform. Our goal is to compute an envelope function to apply to the voltage waveform, such that the natural current waveform is shaped to achieve this desired waveform without feedback control. From the previous section, we saw that the natural current waveform in response to a sinusoidal voltage step input $v_{src}(t)=V_0\sin(\omega_0 t)\,\theta(t)$, at the resonant frequency of the LCR circuit is given by

$$i_{nat}(t)\approx\frac{V_0}{R}(1-e^{-\alpha t})\sin\left(\omega_0 t\right)\theta(t)$$

for $\alpha\ll\omega_0$. This current response corresponds to system with step response $H_{nat}(s)$. We would thus like to compute $v_{env}(t)$ such that the current output of the LCR circuit matches that of the desired current waveform. In the Laplace domain, this relation can be expressed as the following identical expressions:

$$V_{env}(s)H_{nat}(s)=V_{src}(s)E(s)H_{nat}(s)=V_{src}(s)H_{des}(s)=I_{des}(s)$$

From this analysis, we can inspect the envelope function:

$$E(s)=\frac{H_{des}(s)}{H_{nat}(s)}=\frac{\gamma}{\alpha}\frac{s^2+2\alpha s+\omega^2}{s^2+2\gamma s+\omega^2}$$

Because the envelope is defined as a multiplication in the Laplace domain, to apply the envelope in the time domain we convolve the computed envelope function with the sinusoidal step function to retrieve the final enveloped voltage function (or simply the inverse Laplace transform of $V_{env}(s)=V_{src}(s)E(s)$):

$$v_{env}(t) = V_0 \frac{\alpha\sqrt{-\gamma^2+\omega^2}\sin(\omega t) - \omega(\alpha-\gamma)\sin\left(\sqrt{-\gamma^2+\omega^2}\,t\right)e^{-\gamma t}}{\alpha\sqrt{-\gamma^2+\omega^2}}\theta(t)$$

Again, under the assumption that $\gamma \ll \omega$, we can approximate the following term as $\sqrt{-\gamma^2+\omega^2} \approx \omega$, simplifying the above to:

$$v_{env}(t) \approx V_0 \frac{\alpha\omega\sin(\omega t) - \omega(\alpha-\gamma)\sin(\omega t)\,e^{-\gamma t}}{\alpha\omega}\theta(t)$$

$$= V_0\left(1 - \frac{\alpha-\gamma}{\alpha}e^{-\gamma t}\right)\sin(\omega t)\,\theta(t)$$

where the approximate function appears as the original sinusoidal step input modulated by an envelope function:

$$e_{approx}(t) = 1 - \frac{\alpha-\gamma}{\alpha}e^{-\gamma t} = 1 - \frac{\tau_\gamma - \tau_\alpha}{\tau_\gamma}e^{-t/\tau_\gamma}$$

where the subscript approx denotes the approximate multiplicative envelope function applied to the original sinusoidal step, which is not equivalent to the inverse Laplace transform of $E(s)$, which is to be convolved with the original input. Note that as $t \to 0$, $e_{approx}(t) \to \gamma/\alpha$, and as $t \to \infty$, $e_{approx}(t) \to 1$. We can glean insights into pulse shaping by inspecting the form of this multiplicative envelope. If we desire the rise time of the LCR current to increase, such that $\gamma > \alpha$ (recall that the time constants for the envelopes correspond to the inverse of $\gamma, \alpha$, such that this is equivalent to $\tau_\gamma < \tau_\alpha$, resulting in a faster rise time with smaller time constant), we require an increase in the initial voltage amplitude. However, this voltage amplitude decays with the time constant $\tau_\gamma$ to the required steady state amplitude corresponding to the proper steady state current amplitude. On the other hand, if we desire to slow down the rise time such that $\gamma < \alpha$, we begin with a lower voltage amplitude that rises to the final amplitude with time constant $\tau_\gamma$.

These intuitions are valuable for implementation in the hardware. To eliminate the rampup as a confounding variable in experiments, we will choose a constant rise time in terms of number of cycles for each resonant condition. Depending on the natural time constants of the LCR circuits in the system, we must be careful to choose a rise time that is achievable based on the voltage limitation of the amplifiers in use. In practice, we will choose to "slow down" the resonant envelopes to the slowest natural LCR time constant, so that for each condition we do not require additional voltage at the beginning of each pulse. This method can generalize to any desired pulse shape, given adequate capabilities of amplifiers used for driving the circuit.

### C. Untuned Pulse Shaping

Neglecting parasitic effects, the coil impedance at the output of the amplifier can be expressed as:

$$Z(\omega) = j\omega L + R$$

where L is the inductance of the coil, and R is the series resistance of the coil. In general, the series resistance of the coil varies with frequency. Upon applying a sinusoidal voltage waveform, as using an amplifier in voltage control mode:

$$v(t) = V_0 \sin(\omega t)$$

the current waveform will also take a sinusoidal response:

$$i(t) = \frac{V_0}{|Z(\omega)|} \sin(\omega t - \angle Z(\omega))$$

where $|Z(\omega)| = \sqrt{(\omega L)^2 + R^2}$, and $\angle Z(\omega) = \tan^{-1}(\omega L/R)$. In the limiting case of $\omega L \gg R$, note that $|Z(\omega)| \approx \omega L$ and $\angle Z(\omega) \approx \pi/2$, resulting in the current waveform:

$$i(t) = \frac{V_0}{\omega L} \cos(\omega t)$$

To reduce the impact of pulse envelope shape on the experimental results, an exponential envelope is applied to the voltage waveform to mimic the dynamics of the second order LCR circuit in the tuned case from the previous section. The voltage waveform from the amplifier is then:

$$v_{env}(t) = V_0 \left(1 - \exp\left(-\frac{t}{\tau_{des}}\right)\right) \sin(\omega t)$$

where $\tau_{des}$ is the desired time constant of the envelope, resembling the natural step response dynamics of the second order LCR circuit in the preceding section (the inverse of $\gamma$ from the previous section). This rampup envelope also alleviates the burden on the amplifiers – rather than desiring full current amplitude at t=0 as in the case without the voltage envelope, the amplifier can accordingly rampup the current waveform. The current then takes the form:

$$i(t) = \frac{V_0}{|Z(\omega)|} \left(1 - \exp\left(-\frac{t}{\tau_{des}}\right)\right) \sin(\omega t - \angle Z(\omega))$$
